# 

**Published:** 1892-10-15

**Authors:** 


**Notices of Births, Deaths, and Marriages,** are inserted in this column at a charge of 2s. 6d. for an announcement not exceeding four lines.

### NOTICE TO CORRESPONDENTS.

All MS., Letters, Books for Review, and other matters intended for the Editor should be addressed The Editor, The Lodge, Porchester Square, London, W.

The Editor cannot undertake to return rejected M.S., even when accompanied by stamped directed envelope.

**Notice to Advertisers and Subscribers.**

All Advertisements, Orders for Copies of The Hospital, and Business Communications should be addressed to The Publisher, *not to the Editor*, The Hospital, 140, Strand, W.C.

The Cost of Subscription, post free, is as follows:—Per week, 2½d.; per quarter, 2s. 6d.; per half-year, 5s.; per annum, 8s. 6d. Foreign:—Per annum, 10s. 8d.; payable, in all cases, in advance.

**"Burdett's Hospital Annual," 1891—1892.**

Third year of publication. 600 pp. Boards, gilt lettering. A most complete guide to British and Colonial Hospitals, Dispensaries, Nursing and Convalescent Institutions, and Asylums. Price 3s. 6d. Published by The Scientific Press (Limited), 140, Strand, W.C.

**NOTICE.—The Index for Vol. XI. (ending March, 1892) is on sale at the Office of this Journal, price 2½d., post free.**

### THE STUDENT OF MEDICINE.

[All communications for this Supplement should be addressed to The Editor of The Hospital, at 140, Strand, with the words, "Students' Column," written in the left hand top corner of the envelope.]

### APPOINTMENTS.

Archer, E. J., M.B., C.M.Dub., appointed, pro. tem., Medical Officer for the Second Sanitary District of the Southampton Incorporation. Bailey, T. C., L.R.C.P., L.M.Edin., M.R.C.S., appointed Medical Officer for the Crewe Sanitary District of the Nantwich Union. Carling, W., M.B., B.C.Camb., appointed House-Surgeon to Guy's Hospital. Carnelley, M., L.R.C.P.Lond., M.R.C.S., appointed Medical Officer for the Gotham Sanitary District of the Bashford Union. Chaplin, T. H. Arnold, M.B., M.R.C.P., appointed Assistant Physician to the City of London Hospital for Diseases of the Chest, Victoria Park. Clarke, Thomas F., M.R.C.S.Eng., L.S.A., appointed Medical Officer for the First District of the Dartford Union. Cowan, Michael W. W., M.B., C.M.Edin,, appointed Medical Officer of the Knightwick District of the Martley Union. Digby, C.A., M.D., B.Ch.Irel., appointed Medical Officer for the Ticehurst Sanitary District of the Ticehurst Union. Francis, Ernest Edward, M.R.C.S., L.S.A., appointed Surgeon to the Assam Bengal Railway. Fraser, Donald Alexander, M.D.Brux., M.R.C.S.Eng., L.S.A., re-appointed Medical Officer of Health for Totnes Borough. Forrester, R. A. P., M.B., C.M.Edin., appointed Health Officer of Wycheproof, Victoria, South Australia. Godson, A. H., M.B., B.C.Camb., appointed House Surgeon to Guy's Hospital. Grigg, William Hughes, M.R.C.S.Eng., L.S.A., re-appointed Medical Officer for the Hockworthy District of the Tiverton Union. Haydon, William Rudall, M.D., M.B., C.M.Glasg., re-appointed Medical Officer for the Washfield District of the Tiverton Union. Hirsch, Charles T. W., M.R.C.S.Eng., L.R.C.P.Lond., L.S.A., appointed Assistant Government Medical Officer at Fiji. Hooper, Marshall, L.R.C.P., L.R.C.S.Edin., re-appointed Medical Officer for the Codner Park Sanitary District of the Bashford Union. Housley, J., M.D.St.And., M.R.C.S., re-appointed Medical Officer of Health for the East Retford Sanitary District. Jewell, J. W. F. M.B.Lond., M.R.C.S., L.R.C.P., appointed House Physician to Guy's Hospital. Kempe, C. M., M.R.C.S., re-appointed Medical Officer of Health for the Port of Shoreham. Kempster, William Hy., M.D.St.And., L.R.C.P.Lond., M.R.C.S.Eng., appointed Medical Officer of Health for Battersea. Landon, E. E. B., M.R.C.S., L.R.C.P., appointed Assistant House Physician to Guy's Hospital. Liesching, Charles Edward, L.R.C.P.Lond., M.R.C.S. Eng., re-appointed Medical Officer for the Sampford Peverell District of the Tiverton Union. Lyle, Henry, M.R.C.S.Eng., appointed Honorary Assistant Surgeon to the Cancer and Skin Hospital, Liverpool. McKay, Joseph B., M.D., R.U.I., appointed Medical Officer for the Fifth District and the Schools of the Wycombe Union. May, Frederick, L.A.H.Dub., re-appointed Medical Officer for the South District of the Stratton Union. Mullalby, W. T., M.D., M.Ch., L.R.C.P.Irel., appointed Health Officer for the Shire of Ballarat, Victoria, Australia. Robinson, Dr., appointed Medical Officer for the Fleetwood District of the Fylde Union. Sheen, A. W., M.B.Lond., L.R.C.P., M.R.C.S., appointed Assistant House Surgeon to Guy's Hospital. Sichel, G.T.S., M.R.C.S., L.R.C.P., appointed Assistant House Surgeon to Guy's Hospital. Thomas, I. D., M.B., B.C.Camb., appointed Assistant House Physician to Guy's Hospital. Ward, Anthony Arthur, L.R.C.P.Lond., M.R.C.S.Eng., appointed Medical Officer for the Christ Church Sanitary District and the Branch Workhouse of the Parish of St. Giles, Camberwell. Williams, G. R.,

### The Hospital Advertiser.

THE STUDENT OF MEDICINE—(*continued*).

L.R.C.P., L.M., L.R.C.S.Irel., appointed Medical Officer for the Seventh Sanitary District of the Depwade Union. Wilson, Arthur C. J., L.R.C.P., L.M. Edin., M.R.C.S.Eng., re-appointed Medical Officer of Health to the Thurlstone Local Board. Young, F. C., M.B., B.C.Camb., appointed House Physician to Guy's Hospital. Young, Richard W., M.R.C.S., L.S.A., appointed Government Medical Officer and Vaccinator for the Lower Clarence River District, New South Wales.

VACANCIES.

The following vacancies are announced this week, the amount of salary in each case being given after the name of the institution:

**Resident Medical Officers.**

Inverness District Asylum, Med'cal Superintendent.

Manchester Clinical Hospital for Women and Children, Park Place, Cheetham Hill Road, Manchester, House Surgeon—Salary £80 per annum, with apartments and board.

Royal Free Hospital, Gray's Inn Road, W.C., Junior Resident Medical Officer, doubly qualified—Board and residence provided.

Royal Hospital for Diseases of the Chest, City Road, E.C., Resident Medical Officer—Appointment for six months. Salary at the rate of £100 per annum, with furnished apartments and board.

**Non-Resident Medical Officers.**

Charing Cross Medical School, Lecturer on Organic Chemistry.—Remuneration a guaranteed minimum of £100 per annum.

City of London Hospital for Diseases of the Chest, Victoria Park, E., Pathologist—Salary 100 guineas per annum.

County Borough of Stockport, Medical Officer of Health for the District of the Borough, and to the Infectious Diseases Hospital, and Surgeon to the Police—Salary £400 per annum.

Huddersfield Infirmary, Honorary Physician.

